# The Stabilisation Potential of Individual and Mixed Assemblages of Natural Bacteria and Microalgae

**DOI:** 10.1371/journal.pone.0013794

**Published:** 2010-11-02

**Authors:** Helen V. Lubarsky, Cédric Hubas, Melanie Chocholek, Fredrik Larson, Werner Manz, David M. Paterson, Sabine U. Gerbersdorf

**Affiliations:** 1 Sediment Ecology Research Group, Scottish Oceans Institute, University of St. Andrews, St. Andrews, Scotland, United Kingdom; 2 Institute of Hydraulic Engineering, University Stuttgart, Stuttgart, Germany; 3 Muséum National d'Histoire Naturelle, UMR BOREA (Biologie des organismes et écosystèmes aquatiques) MNHN-CNRS-UPMC-IRD, Département Milieux et Peuplements Aquatiques (DMPA), Paris, France; 4 Swedish Board of Fisheries, Gothenburg, Sweden; 5 Institute for Integrated Natural Sciences, University Koblenz - Landau, Koblenz, Germany; University of Wisconsin-Milwaukee, United States of America

## Abstract

It is recognized that microorganisms inhabiting natural sediments significantly mediate the erosive response of the bed (“ecosystem engineers”) through the secretion of naturally adhesive organic material (EPS: extracellular polymeric substances). However, little is known about the individual engineering capability of the main biofilm components (heterotrophic bacteria and autotrophic microalgae) in terms of their individual contribution to the EPS pool and their relative functional contribution to substratum stabilisation. This paper investigates the engineering effects on a non-cohesive test bed as the surface was colonised by natural benthic assemblages (prokaryotic, eukaryotic and mixed cultures) of bacteria and microalgae. MagPI (Magnetic Particle Induction) and CSM (Cohesive Strength Meter) respectively determined the adhesive capacity and the cohesive strength of the culture surface. Stabilisation was significantly higher for the bacterial assemblages (up to a factor of 2) than for axenic microalgal assemblages. The EPS concentration and the EPS composition (carbohydrates and proteins) were both important in determining stabilisation. The peak of engineering effect was significantly greater in the mixed assemblage as compared to the bacterial (x 1.2) and axenic diatom (x 1.7) cultures. The possibility of synergistic effects between the bacterial and algal cultures in terms of stability was examined and rejected although the concentration of EPS did show a synergistic elevation in mixed culture. The rapid development and overall stabilisation potential of the various assemblages was impressive (x 7.5 and ×9.5, for MagPI and CSM, respectively, as compared to controls). We confirmed the important role of heterotrophic bacteria in “biostabilisation” and highlighted the interactions between autotrophic and heterotrophic biofilm consortia. This information contributes to the conceptual understanding of the microbial sediment engineering that represents an important ecosystem function and service in aquatic habitats.

## Introduction

Biofilms represent the dominant microbial life in many aquatic systems and drive a number of important “ecosystem services” such as nutrient recycling, biodegradation and pollutant retention [1]. In recent years it has been shown that benthic biofilms can also act as a protective layer at the sediment surface that can significantly influence erosion and deposition of sediment particles [2]. The major mechanism of this microbial “biostabilisation” is through the production of a matrix of extracellular polymeric substances (EPS), a heterogeneous mixture of polysaccharides, proteins, nucleic acids, lipids and humic acids [3], secreted by biofilms cells. While a range of meio- and microorganisms secrete EPS, most studies have focussed on benthic microalgae as the main EPS producers, with carbohydrates as their main product [2], [4], [5]. Positive correlations between sediment stability and microalgal biomass and/or EPS carbohydrates have often been described, most of which were from marine intertidal sites, and highly site-specific [6], [7], [8]. Nevertheless, due to the microalgal influence on the structure and behaviour of sedimentary habitats, they have been put forward as important “ecosystem engineers” [9]. While biostabilisation by microalgae has been researched extensively in the marine habitat, the ubiquitous heterotrophic bacteria have largely been ignored, even in conceptual models. Heterotrophic bacteria have been mainly regarded as decomposers of the organic matrix [10] and as acting in response to microalgal exudates [11], [12]. However, bacteria also produce copious amounts of EPS, as recognised from biomedical, biotechnological or industrial studies [13], [14], [15]. Pioneering work on the entrainment of a clay-water suspension by Dade et al. [16] and on the stability of experimentally-derived biofilms by Leon-Morales et al. [17] indicated significant effects of bacterial exopolymers on the substratum. These studies inspired our recent work which has shown that natural benthic bacterial assemblages from estuarine areas significantly stabilized a test substratum, far exceeding our expectations, as based on the limited literature [18], [19]. The former work on the sediment stabilisation potential of microalgae appears in a new light, since the natural “microalgal mats” investigated were certainly not devoid of heterotrophic bacteria. Hence, the question of the functional role and origin of EPS in microbial mats requires further interpretation and can initially be addressed by separate studies of the engineering potential of prokaryotic and eukaryotic assemblages.

There is evidence that the co-existence of bacteria and microalgae might be of mutual advantage mainly in terms of nutrient recycling [10], [20]. Some microalgal species depend on association with certain bacteria groups [“satellite bacteria”, 21], and in some pelagic diatoms, the presence of specific bacteria is crucial for their growth and EPS secretion [22]. Bruckner [11] et al. showed that the monomer composition of microalgal EPS carbohydrates varied along with the presence of different bacterial groups. On the other hand, some microalgae species suppress bacteria by producing polyunsaturated aldehydes that have strong bactericidal effects [23], [24]. Bacteria can also influence microalgal growth and EPS secretion through the release of specific algicidal compounds [25], [26]. There is evidence that these bacteria-microalgae interactions are highly species-specific and help to shape the composition of the biofilm assemblages [27], again with possible implications for EPS secretion and sediment binding. Presumably, the various bacteria-microalgae interactions are strongly driven by abiotic and biotic conditions both within and outside the biofilm. For instance, external nutrient addition caused shifts within the natural microbial assemblage which influenced EPS concentration, EPS composition and sediment stability [18], [19]. Still, the mechanisms and species interactions inducing these shifts in biofilms are far from understood and nutrients are not the only influential environmental variable.

The present paper compared the individual and combined engineering capability of natural heterotrophic bacterial assemblages (“B”), axenic autotrophic microalgal/diatom assemblages (“D”) and mixed assemblages of both (“BD”) in terms of EPS secretion and substratum stabilisation. The adhesive capacity of the surface as well as the resistance to erosion, both proxies for sediment stability, were monitored regularly by Magnetic Particle Induction (MagPI) and Cohesive Strength Meter (CSM), respectively, and related to microbial growth (bacterial cell numbers, bacterial dividing rate, microalgal biomass) and EPS secretion (concentrations/composition of carbohydrates and proteins). It was hypothesized that the coexistence of bacteria and microalgae might show synergistic effects on EPS secretion, cell growth and the net engineering potential.

## Results

### Microphytobenthos composition

In the mixed assemblage (bacteria + diatoms, BD), diatoms of the genera *Achnanthes*, *Caloneis*, *Navicula* and *Nitzschia* were the intial colonizers of the substratum at the beginning of the experiment (day 1). While the large species *Achnanthes longipes* and *Caloneis amphisbaena* were dominant, the majority of species were represented by the genus *Navicula* (*N. cinta, N. digitoradiata, N. flanatica N. gregaria N. crytocephala, N. perminuta/diserta N. phyllepta N. salinarum*) and *Nitzschia (N. epithemioides, N. frustulum, N. hungarica, N. sigma*). Over time, smaller species, such as *Navicula,* became increasingly dominant together with *Nitzschia* and *Cymbella* species. After 4 weeks, only small *Navicula* species remained in the culture. In the diatom assemblage (D), treated with antibiotics to inhibit bacterial colonization, the species composition was quite similar to the mixed assemblage with *Achnanthes*, *Cylindrotheca*, *Cymbella*, *Navicula* and *Nitzschia* species present but smaller *Navicula* species were dominant from the beginning. *Achnanthes*, *Cymbella*, and *Nitzschia* species were characteristic for this treatment for about 3 weeks. By the end of the experiment, only small *Navicula* species remained.

Most of the diatom species were typically from poly- and hypertrophic environments, except for some species of Achnanthes and Cymbella that require mesotrophic conditions. Although the benthic diatom community was isolated from natural sediments, species richness seemed less diverse as compared to the natural habitats.

### Bacterial assemblages

The proportion of the active cells, as determined by EUB mix, was higher at the start of the incubations for the pure bacterial assemblage (B, 58%) as compared to the mixed assemblage (BD, 38%); however at the end of the experiment the proportion of active cells was similar for both treatments (54%, B and 55%, BD), indicating that most of the bacterial community was metabolically active at the relevant sampling time. In the control measurements (C) as well as in the diatom assemblage (D), hybridization with oligonucleotide probes was below levels of detection.

The application of domain, phylum, and subphylum specific oligonucleotide probes (Table 1) revealed that gram-negative Proteobacteria dominated the samples, while gram-positive Actinobacteria were less than 1% of the total bacteria (Table 2). In the mixed assemblage, the Alphaproteobacteria accounted for 18%, the Betaproteobacteria for 35%, the Gammaproteobacteria for 15%, the Delta-subclass for 5% and the Cytophaga Flexibacter Subphylums for 15%. Over time, a noticeably shift was determined within the assemblage: while the Alphaproteobacteria increased to 20%, the Betaproteobacteria decreased to 18%, and Sulphate deoxidizer/Delta-subclass decreased below detection limits. The Actinobacteria accounted for less than 1% and were thus negligible. The pure bacterial assemblage showed similar proportions of the subphyla (Alphaproteobacteria 10%, Betaproteobacteria 30%, Gammaproteobacteria 10%, Cytophaga/Flexibacter 13%), but the Delta-subclass could not be detected. Over time, Alphaproteobacteria increased (to 12%) and the Betaproteobacteria decreased, but to a much lesser extend (to 25%) as compared to the mixed assemblage. Noticeably different to the BD treatment was the increase in Gammaproteobacteria (to 25%) and Cytophaga/Flexibacter (to 18%) over time. Like in the mixed assemblage, the gram-positive Actinobacteria were present at low relatively abundance of <1% (Table 2).

**Table 1:** Oligonucleotides used in this study (^a^ Probe nomenclature as described by Alm et al. (1996).

Target organisms	Oligonucleotide^a^ Common name	Sequence (5′–3′)	%FA^b^	Reference
*Bacteria*	S-D-Bact-0338-a-A-18 EUB338	GCTGCCTCCCGTAGGAGT	0–50	Amann *et al., (*1990)
*Plantomycetales*	S-D-Bact-0338-b-A-18 EUB338 II	GCAGCCACCCGTAGGTGT	0–50	Daims *et al.,* (1999)
*Verrucomicrobiales*	S-D-Bact-0338-c-A-18 EUB338 III	GCTGCCACCCGTAGGTGT	0–50	Daims *et al., (*1999)
*Alphaproteobacteria*	S-Sc-aProt-0019-a-A- ALF968	GGTAAGGTTCTGCGCGTT	35	Neef, 1997
*Betaproteobacteria*	L-Sc-bProt-1027-a-A-17 BET42a	GCCTTCCCACTTCGTTT	35	Manz *et al.,* (1992)
*Gammaproteobacteria*	L-Sc-gProt-1027-a-A-17 GAM42a	GCCTTCCCACATCGTTT	35	Manz *et al.,* (1992)
*Actinobacteria*	S-P-HGC-1901-a-A-18 HGC69a	TATAGTTACCACCGCCGT	25	Roller *et al.,* (1994)
*Desulfobacterales, Desulfuromonales, Syntrophobacterales, Myxococcales*	S-F-Srb-0385-b-A-18 (SRB385Db)	CGGCGTTGCTGCGTCAGG	35	Rabus et al., (1996)
*Cytophaga-Flavobacterium group of Bacteroidetes Flavobacteria, Bacteroidetes & Sphingobacteria*	S-P-CyFla-0319-a-A-18 CF319a	TGGTCCGTGTCTVAGTAC	20	Manz *et al.,* (1996)

**Table 2:** Percentage of the specific bacterial groups (marked by the oligonucleotide probes named on the left) of the total eubacterial counts; given for the treatments bacteria and diatoms (BD) as well as bacteria (B) for the beginning (1) and the end (2) of the experiment.

	BD, 1 FA (%)	BD, 2 FA (%)	B, 1 FA (%)	B, 2 FA (%)
ALF968	18	20	10	12
BET42a	35	18	30	25
GAM42a	15	15	10	25
HGC69a	<1	-	-	<1
SRB385Db	5	-	-	<1
CF319a	15	15	13	18

### Microbial biomass, cell numbers and growth rate

**Figure 1:**
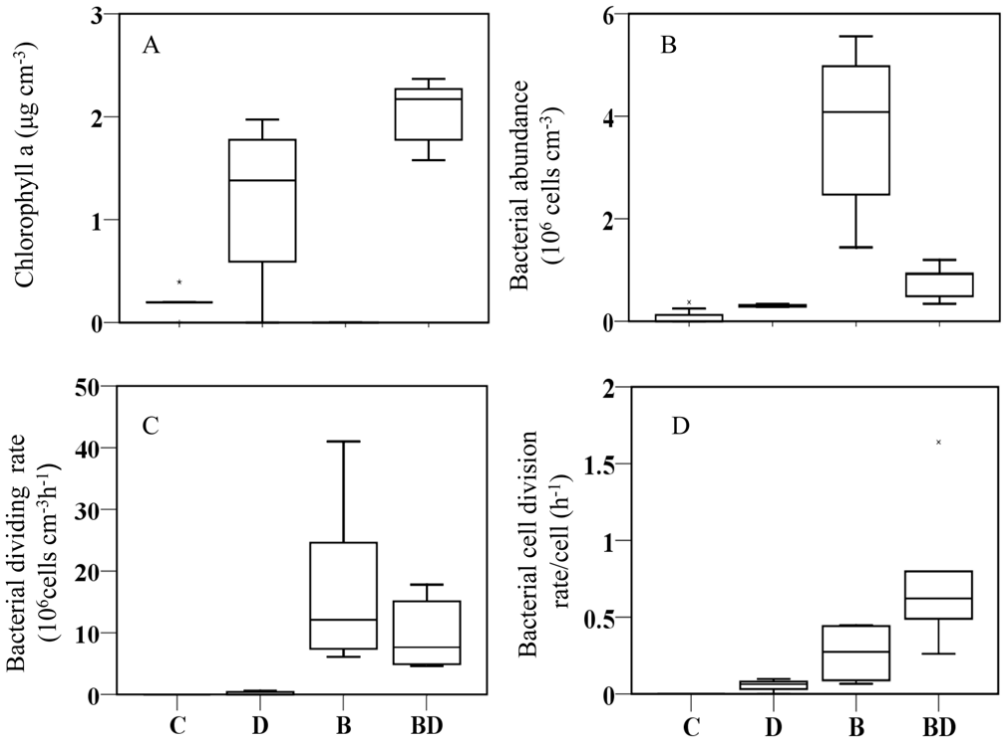
Mean values of the different treatments: mixed assemblages (BD), diatoms (D), bacteria (B), control (C). A. chlorophyll *a* (n = 21). B. bacterial cell numbers (n = 24). C. bacterial division rates (n = 18). D. bacterial specific division rates (n = 18).

The chlorophyll *a* (Chl *a*) and pheophytin concentrations were significantly different between the treatments for most of the sampling days (Kruskal-Wallis (χ^2^) test (KW), p<0.05). Chl *a* concentrations in the mixed treatment ranged between 1.5 and 2.17 µg cm^−3^ and were significantly higher than in the axenic microalgal assemblages (Fig. 1A) with values ranging between 1.38 and 1.97 µg cm^−3^ (for example, day 14: KW, χ^2^ = 6.77 df = 2, p<0.05, with post-hoc Student-Newman-Keuls (SNK) test).

Like the microbial biomass, the bacterial cell numbers determined by flow cytometry significantly differed between the treatments on most of the days (KW, p<0.05). The bacterial cell numbers in the treatment B and BD varied between 1.44×10^7^ and 5.56×10^7^ cells cm^−3^ as well as 0.34×10^6^ and 1.19×10^7^ cells cm^−3^, respectively (Fig. 1B). Thus, the bacterial cell numbers were significantly higher in the pure bacterial culture (for example, day 14: KW, χ^2^ = 3.8, df = 3, p<0.05, with post-hoc SNK test).

Based on the calculated [*methyl-* ^3^H] thymidine incorporation, there was no significant difference for bacterial division rate between the bacterial and mixed assemblages (Fig. 1C). Like the bacterial cell numbers, the bacterial division rates were negligible in the controls and in the axenic diatom assemblage. The specific rate of bacterial division per cell per hour can be calculated by dividing the division rate of the bacterial community (cells cm^−3^ h^−1^) by the bacterial cell numbers (cells cm^−3^). The specific rate of bacterial division was significantly higher for BD as compared to B (Fig. 1D); especially on day 3 (BD 18.2 times higher than B, KW, χ^2^ = 6.2 df = 2, p<0.05, with post-hoc SNK test).

There was no significant correlation between the bacterial cell division rates and bacterial cell numbers in the bacterial treatment or in the mixed assemblage. Despite ongoing growth of microalgae and bacteria, no significant relationships between chlorophyll *a* as a proxy for microalgal biomass and the bacterial cell numbers or bacterial division rates could be determined within the mixed assemblage.

### Changes in EPS components

**Figure 2:**
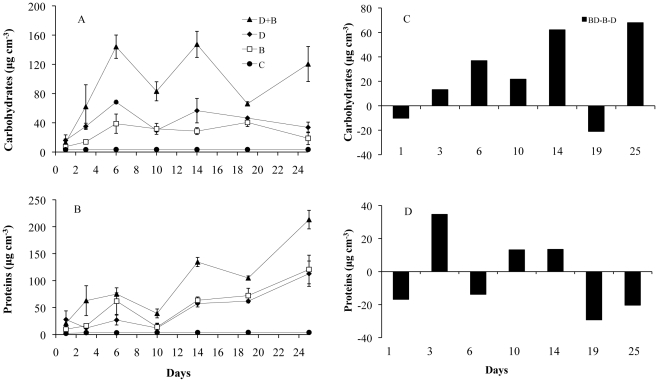
Mean values of EPS concentrations and their relative assessment between treatments. A–B: Mean values (n = 3 per treatment, based on n = 3 replicates per box ± SE) of EPS concentrations in the treatments bacteria and diatoms (BD, ▴), diatoms (D, ♦), bacteria (B, □) and controls (C, •) for carbohydrates (A) and proteins (B). C–D: The EPS concentration of the mixed cultures (BD) relative to the contribution of the single cultures (B and D) such that the value “[BD]-[B+D]” is reported for carbohydrates (C) and proteins (D). Where the production of carbohydrate or protein from mixed cultures (BD) exceeds that of the added single cultures (B and D) the value is positive (synergistic effect) and vice versa (inhibitory effect). If the added values of the single cultures exactly equals the mixed cultures then there is an additive effect.

Over time, the colloidal EPS carbohydrate concentrations increased in all treatments to a maximum on day 14 (Fig. 2A, Table 3), but the increase was most pronounced for the mixed assemblage. The carbohydrate concentrations varied between 13–147.3 µg cm^−3^, 7.3–40.5 µg cm^−3^ and 15.9–56.6 µg cm^−3^ for BD, B and D, respectively (Fig. 2A) with significantly different means in the treatments for all sampling dates except at the beginning of the experiment (KW, p<0.05). The carbohydrate concentrations were significantly higher in BD as compared to D and B (for example, day 14: KW, χ^2^ = 9.66, df = 3, p<0.05, followed by post-hoc SNK test) (Fig. 2A, Table 3). The treatments B and D were not significantly different from each other. The controls showed negligible concentrations of EPS carbohydrates.

**Table 3:** Differences between the first day of sampling (day 1) and day 14 where most of the variables showed their maximum value as well as differences between the given treatments (mixed: BD, Bacteria B, Diatom D); both times expressed as quotient/factors for EPS carbohydrates, EPS proteins, MagPI and CSM.

Factors		*Carbohydrates*	*Proteins*	*MagPI*	*CSM*
**Between day 1–14**	**B**	5.5	6.4	3.4	4
	**D**	3.6	2.1	2.6	2.8
	**BD**	11	6.4	2.9	1.8
**Between treatments**	**BD/B**	5.1	1.7	1.4	2.6
	**BD/D**	2.6	1.9	2.5	4.1
	**B/D**	0.714	-	1.7	1.3

The pattern of the water–extractable protein concentrations over time was similar to that of the carbohydrates, with an increase towards day 14 in all treatments (Fig. 2B, Table 3). The protein concentrations for the treatments BD, B and D varied between 20.9–213.1 µg cm^−3^, 9.8–120.6 µg cm^−3^ and 27.8–112.8 µg cm^−3^, respectively (Fig. 2B) with significantly different means in the treatments for most of the sampling dates (KW, p<0.05). The protein concentrations in the treatment BD were significantly higher than in the treatments B and D (for example, day 14: KW, χ^2^ = 9.67, df = 3, p<0.05, followed by post-hoc SNK test). The treatments B and D were not significantly different from each other. The EPS proteins in the controls were below detection limits.

To explore possible inhibitory, additive or synergistic effects by the liaison of bacteria and microalgae, the amount of EPS produced in each single assemblage (B and D) was assessed relative to the amount of EPS produced in the mixed assemblage ([BD]-[B+D], Fig. 2C and D). Where the result is close to zero, EPS production by B and D is additive with respect to BD, while a negative value suggests either reduced EPS production or enhanced EPS recycling in the mixed assemblage (inhibitory effect). A strongly positive value for the relationship would suggest synergy in the mixed culture. For EPS carbohydrates, the value was strongly positive for most of the sampling days suggesting a synergistic effect (Fig. 2C). The results in terms of EPS protein production were more equivocal with a balance in response across the sampling dates (Fig. 2D).

A strong positive correlation was determined between EPS colloidal carbohydrates and EPS colloidal proteins (Pearson correlation coefficient, r = 0.607, n = 78, p<0.001). The colloidal carbohydrates and proteins showed a significant positive relation to microalgal biomass (r = 0.385, n = 56, p<0.001 and r = 0.310, n = 57 p<0.01, respectively) as well as to the bacterial cell numbers (r = 0.649, n = 18, p<0.01 and r = 0.518, n = 18, p<0.01, respectively).

### The stability of the substratum

**Figure 3:**
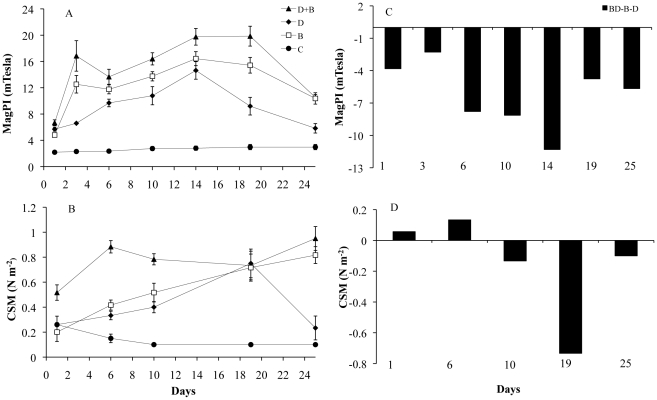
Mean values of MagPI and of CSM measurements and their relative assessment between the treatments. A. Mean values (n = 6) of MagPI over the course of the experiment. B. Mean values (n = 6) of CSM measurements over the course of the experiment. The different treatments were bacteria and diatoms (BD, ▴), diatoms (D, ♦), bacteria (B, □) and controls (C, •). Substratum stability by the mixed BD treatment relative to the stability of the single B and D treatments is given for MagPI (C) and CSM (D). Where the stability created by the mixed culture (BD) exceeds that of the added single cultures (B and D), the value is positive (synergistic effect) and vice versa (inhibitory effect). If the added values of the single cultures equals the mixed cultures then the effect measured is additive.

**Figure 4:**
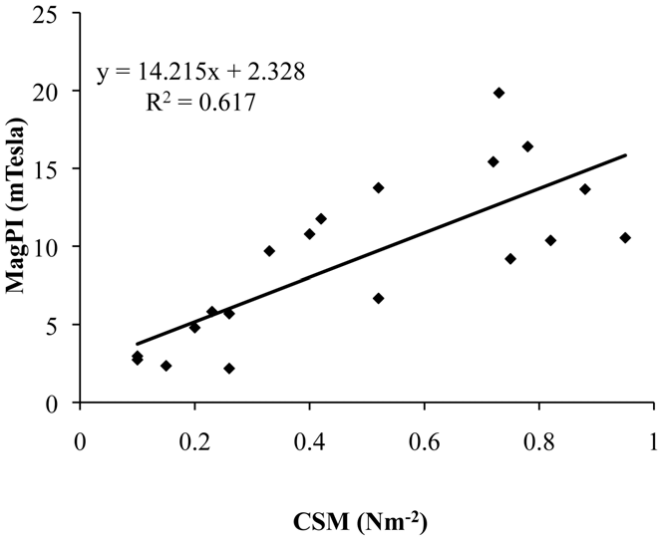
Relationship between MagPI (mTesla) and CSM (Nm^−2^).

The surface adhesion of the substratum as determined by MagPI increased for all treatments over time to a maximum value on day 14 (Fig. 3A, Table 3). Cohesion of the substratum as indicated by CSM increased continuously for all treatments (Fig. 3B, Table 3) over the 4 weeks. The control treatments (C) did not show any significant changes in adhesion/stability over the 25 d of the experiment. There was a significant difference in the means of the treatments for the surface adhesion and cohesion (p<0.05) for all dates except at the beginning of experiment. The mixed assemblage (BD) showed the highest surface adhesion of the sediment followed by the bacterial culture (B) and finally, the diatom biofilms (D). The CSM measurements confirmed the MagPI results with significantly higher sediment surface stability in treatment BD followed by B and D (for example, day 24: KW, χ^2^ = 10.2., df = 3, p<0.05, followed by a post hoc SNK test). There was a strong linear relationship between CSM (erosion threshold) and MagPI (surface adhesion) (Pearson correlation coefficient: r = 0.785, n = 20, p<0.001, Fig. 4).

In order to visualize possible additive/synergistic effects of bacteria-diatom assemblages, this time for sediment stability, their absolute value of adhesion was compared to the values for the pure bacterial and diatom cultures ([BD]-[B+D], Fig. 3C and D). There was a stronger case for interference in the mixed assemblage since the results were much lower than would be expected from the additive effects of the two cultures B and D, as was particularly evident for surface adhesion as determined by MagPI (Fig. 3C and D).

### Relation between biological variables and surface adhesion and stability

**Figure 5:**
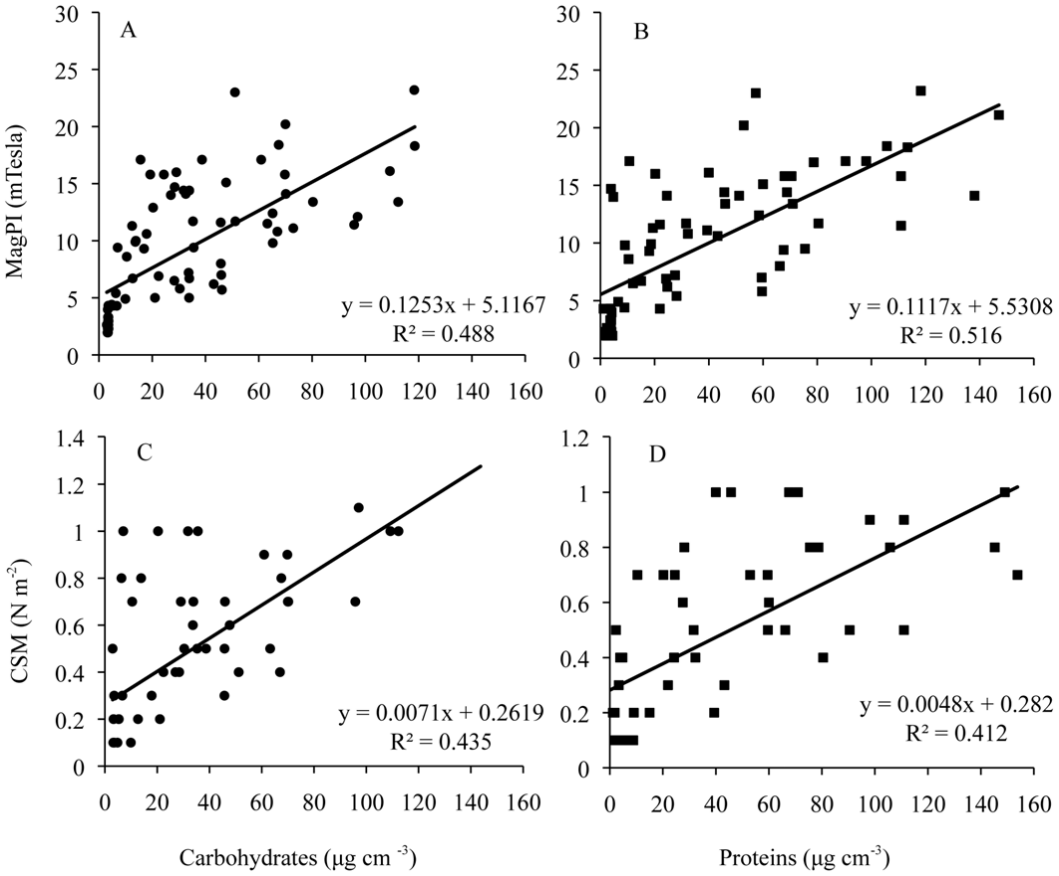
Relationships between sediment stability (MagPI, CSM) and EPS components. A–B. The relationships between surface adhesion (MagPI) and EPS carbohydrates and proteins concentrations. C–D. The relationships between substratum stability (CSM) and EPS carbohydrates and proteins concentrations.

There was a strong positive relationship between sediment stability measurements and chlorophyll *a* concentrations (MagPI: r = 0.395, p<0.001; CSM: r = 0.501, p<0.001). Similarly, EPS carbohydrates concentrations were highly significantly correlated with MagPI and CSM measurements for all treatments. The same applied for the relation of EPS proteins concentrations to adhesion (MagPI) and cohesion (CSM) of the surface for B and BD, while for D the relationships were not significant (Fig. 5, Table 4).

**Table 4:** Pearson's correlation coefficients between surface adhesion (MagPI) as well as substratum stability (CSM) and EPS carbohydrates as well as EPS proteins in the different treatments.

Treatments	Techniques	Carbohydrates			Proteins		
Diatom	MagPI	0.882	17	***	−0.189	21	
	CSM	0.869	11	***	0.321	15	
Bacteria	MagPI	0.861	15	***	0.770	14	**
	CSM	0.753	9	*	0.902	10	***
Bacteria+ Diatom	MagPI	0.706	15	**	0.741	15	**
	CSM	0.617	12	*	0.494	12	*

The significance levels are the following: *** p<0.001. ** p<0.01. * p<0.05.

## Discussion

### Substratum stabilisation by microbial assemblages from estuarine sediments

This study has shown impressive bio-stabilisation of non-cohesive material by microbial assemblages, as determined by Magnetic Particle Induction (MagPI) and Cohesive Strength Meter (CSM). These devices determine slightly different surface properties of the test bed. With MagPI, an increase in adhesion (a proxy for particle capture potential and interface stability) was determined from day 1 and this increased with time in all microbial assemblages. MagPI does not require the erosion of the surface and therefore is a repeatable, sub-critical stress measurement with a high sensitivity that has been shown suitable for measuring the surface properties of young, developing biofilms. The CSM is a well-established device to measure erosion resistance; it requires bed failure and can operate over a range of values beyond that of most linear flumes. The CSM is not designed to mimic the processes of natural erosion since the eroding pressure is perpendicular to the bed but provides an accepted relative measure of surface stability. It also requires a surface that has some initial resistance to erosion or the lightest jet pulse causes a 10% reduction in transmission, and therefore it is not as sensitive as MagPI for highly unconsolidated systems. However, these devices were found to complement each other, increasing the range of measurements that could be made and showed a strong correlation in the overlapping portion of the data (R^2^ = 0.62, p<0.001).

### The individual and combined engineering capability of microbial assemblages

The comparison of pure bacterial, axenic microalgal and mixed (bacteria + microalgae) assemblages was designed to provide insights into the individual and combined functional capacity of the heterotrophic and autotrophic biofilm components in terms of substratum properties. While this is a limited suite of measurements, they demonstrate the functional development of these assemblages in a new light. Bacterial assemblages stabilised the substratum significantly more than axenic microalgal assemblages (x 2). This work supported earlier findings [19] but are in contrast to most of the literature [28], [29], where the contribution of bacteria to sediment stabilisation is usually regarded as less significant or even negligible as compared with diatom assemblages. Separation of the influence of component assemblages of bacteria and diatoms in nature is problematic. Our approach was to use assemblages derived from natural systems but manipulated to create the segregation of bacteria and diatoms. We used a mixture of antibiotics to inhibit bacterial growth and we understand there are some potential problems with this. Chloramphenicol has been reported to suppress the growth of microalgae in general and diatoms in particular [30], [31]. It is also known that some microalgae, among them diatoms, require an association with bacteria to thrive [11], [22], [25], [32], [33]. In this study, the microalgal biomass was significantly lower in the axenic diatom assemblage (D) as compared to the assemblage associated with bacteria (BD) which may be an indication of antibiotic treatment effects or the influence of bacteria/diatom association. In contrast, the bacterial growth in the pure culture without microalgae was good.

It was first hypothesized that the grouping of bacteria and diatoms in the mixed assemblages might result in synergy in community EPS secretion and therefore substratum stabilisation. The first of these concepts is supported by the data in terms of EPS carbohydrate production but not for EPS protein production. However, the synergism in EPS carbohydrate was not reflected in surface stability by either method of determination (MagPI, CSM). While the adhesive capacity and the cohesion of the test surfaces were significantly higher in the mixed assemblage, the differences against the pure cultures were less than expected. This may be because the shape of the relationship between EPS concentration and surface stability is not linear and may reach an asymptote as EPS increases. This makes logical sense since by adding more EPS the strength of the surface cannot increase beyond the fundamental binding capacity of the polymer. The improved binding by the mixed culture may reflect the contribution of different types of EPS with varied properties and the nature of the micro-spatial arrangement of the EPS deposited by bacteria (largely attachment to grains) and diatoms (for locomotion) (Fig 4).

It is often suggested that diatom growth and EPS secretion is promoted by nutrient recycling by bacteria [20], [22], [32], [34]. Over the first 10 days of the experiment, the greater growth of microalgae in the natural assemblage, as compared to the axenic microalgal culture, seemed to support this possibility. However, with time, the microalgal biomass decreased to comparable levels in both treatments. Furthermore, the microalgal community composition was quite similar over time in both biofilms and thus gave no support to the suggestion of selection or inhibition of microalgae by these bacteria. The natural and axenic microalgal assemblages were both dominated by typical poly- to hypertrophic species found in fresh-brackish waters. In the last week of the experiment, species diversity declined similarly in both biofilms until only small *Navicula* species remained suggesting laboratory conditions were not ideal, supporting earlier work on diatom assemblages in laboratory systems [35]. Surprisingly, the bacterial cell numbers, along with the bacterial dividing rates, were significantly lower in the mixed assemblage as compared to the pure bacterial culture. In the literature, it is reported that bacteria development is often concomitant with benthic microalgae [36] and they adapt quickly to the different organic microalgal exudates with substrate-specific responses regarding enzyme activity, usually resulting in compositional shifts and stimulated bacterial growth and metabolic activity [12], [21]. However, the bacteria consortia that developed in our systems did not seem to profit from the presence of diatoms. There is a possibility that the diatoms actively suppressed the bacteria since it is known that marine bacteria are very sensitive to polyunsaturated aldehydes (PUAs) produced by a range of microalgae species [23], [24]. This possibility requires further study in benthic systems. However, it is perhaps more likely that we observed a selection/adaptation process as the natural microbial biofilms adapted to culture conditions and populations capable of co-existing or exploiting algal/bacterial species were promoted, as has been shown for floodplains and estuaries [12], [27]. Indeed, the bacterial community showed pronounced compositional shifts with the presence of diatoms during the experiment. While the gram negative Proteobacteria constituted the majority of the bacterial community, the percentage of α, β, γ - Proteobacteria changed over time. Members of α - Proteobacteria as well as the *Cytophaga-Flavobacterium-Bacteroides* (CFB) phylum have been identified as “satellite bacteria” for marine diatoms [21]. Interestingly, α - Proteobacteria were more prominent in the mixed assemblage than in the bacterial culture, although the absolute increase over time was similar in the two relevant treatments. However, the hybridization to the CFB phylum did not increase over time in the mixed assemblage. β - Proteobacteria decreased in both treatments, but this was more pronounced in the natural assemblage where the presence of diatoms might have been a factor. The γ - Proteobacteria increased solely in the bacterial assemblages and remained unchanged in the mixed biofilm, and thus seem to have a lesser prominence in the presence of diatoms. Hence, the composition of the bacterial assemblage was responsive to the presence of diatoms.

### The EPS Matrix – key to substratum stabilisation?

It has generally been reported that diatoms secrete mainly polysaccharide EPS while bacteria secrete a greater proportion of proteins in their EPS [e.g.[3],[37]]. This is supported by the significantly higher carbohydrate concentrations in the axenic microalgal assemblage as opposed to the bacterial biofilm. Despite this, the stabilisation effect of the bacterial assemblage was significantly higher than in the microalgal biofilms, although the EPS protein concentrations were quite similar. This strongly suggests that EPS quantity *per se* cannot be predictive of substratum stabilisation. The ecological function of the microbial EPS secretion has to be considered: for instance, bacteria attach firmly to a substratum with the help of EPS while diatoms secrete EPS for locomotion [38]. Thus, it seems logical to suggest that the EPS secreted by bacteria and diatoms must differ in their characteristics and mechanical properties. This variation in properties might explain the unexpectedly greater stabilisation capability of bacterial cultures as compared to the axenic diatom cultures. These finding also support earlier work suggesting that proteins play a more significant role in substratum adhesion/cohesion than previously thought [18], [19]. Hydrophobicity, surface charges (Zeta potential) and the free energy of microbial cell surroundings/EPS are crucial factors controlling the “first kiss”, the attachment of a microbe to a surface [e.g. 39]. Proteins play a significant role in this first adhesion [15], [40], but also contribute towards the binding strength within the developing EPS matrix. This has been demonstrated for marine aggregates, where the incorporation of free protein particles significantly increased stability [41]. If EPS proteins interact with carbohydrates, they can form a resilient matrix similar to an epoxy resin [42]. The degree of bonding also depend on the lengths of the polymers involved and the degree to which they branch [42], [43].

**Figure 6:**
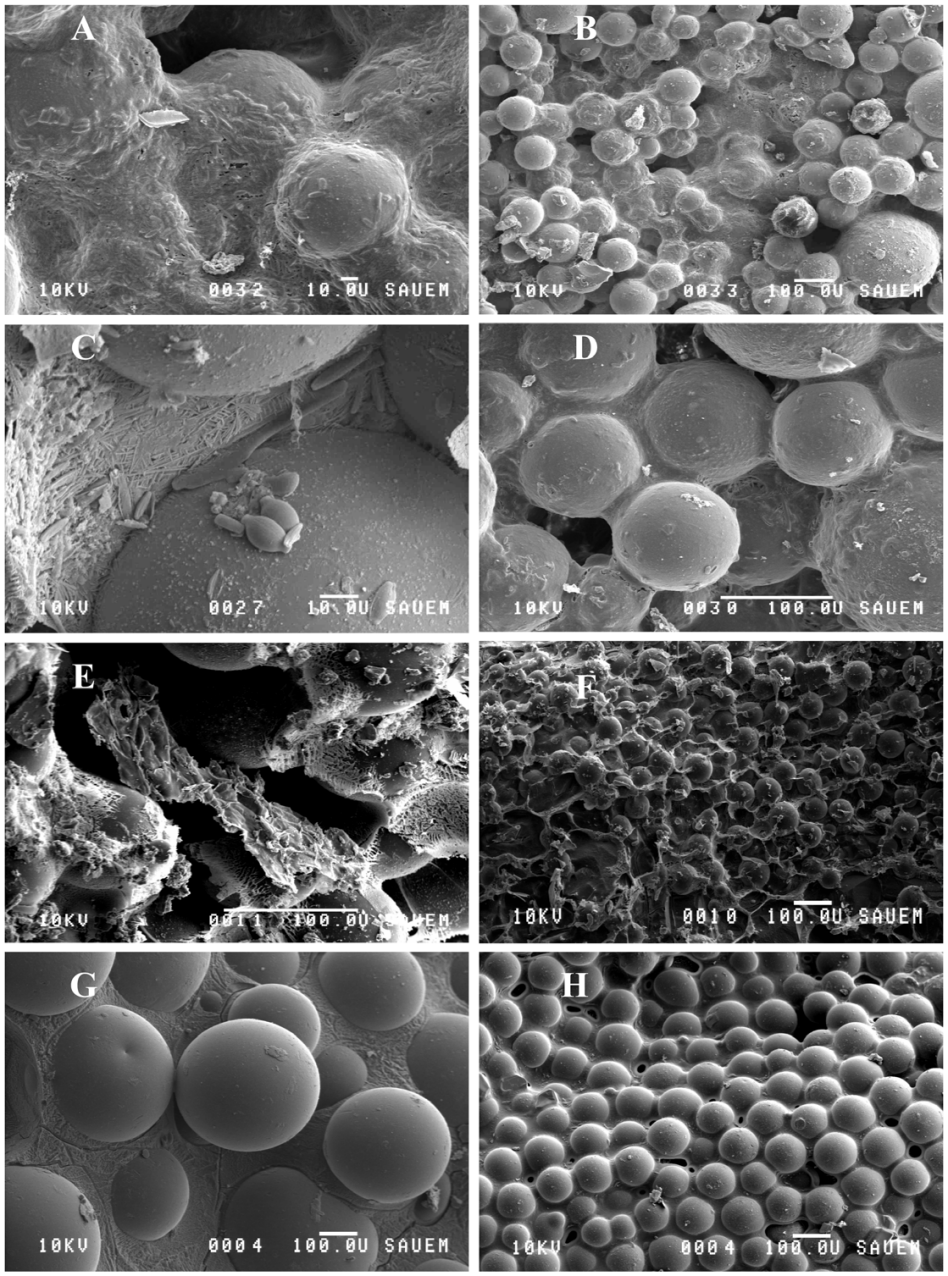
Low-temperature scanning electron microscope images using different magnifications. A–B. The mixed assemblages bacteria + diatom. C–D. The diatom treatment. E–F. The bacteria treatment. G–H. The control substratum. Frozen water (ice) on the surface produces a solid matrix around the glass beads in the controls. In the other treatments with microorganisms, the EPS matrix is visible, heavily covering the glass beads and permeating the intermediate pore space.

Neither carbohydrates nor proteins are exclusively linked to microalgae or bacteria and their proportion might not always be as suggested in the literature. Consequently, EPS carbohydrates and EPS proteins in the mixed assemblage were significantly and positively correlated to microalgal biomass *and* bacterial cell numbers. In addition, the characteristics of one particular EPS component, carbohydrates or proteins, most likely differs between the heterotrophic and autotrophic producers. The greatest functional effect of natural assemblages in terms of substratum stabilisation coincided with significantly higher quantities of microbial produced colloidal EPS carbohydrates and EPS proteins (Fig. 6).

Although our initial hypothesis of synergistic effects in a combined prokaryotic and eukaryotic biofilm community in terms of stability was not supported, the functional capacity for adhesion and cohesion by the liaison between bacteria and microalgae was impressive. This biostabilisation is an important “ecosystem service” since it affects processes beyond the biofilm such as nutrient fluxes, pollutant retention and sediment erosion/transport.

### Conclusions

The stabilisation of the substratum by estuarine microbial assemblages was due to the secreted EPS matrix, and both EPS concentrations (quantity) and EPS components (quality) were important. In this context, the EPS proteins seem to play a crucial role for adhesion/cohesion of the substratum. Bacterial assemblages had a significantly higher stabilisation potential as compared to the axenic microalgal cultures. The explanation is probably in the conformation of the polymeric matrix and may reflect the functional roles (attachment, movement) that the EPS provides. The mixed assemblages were more stable than either community on its own and this suggests both assemblages have an important role in substratum stabilisation and are more effective together. The tendency in the literature to exclude the contribution of bacterial EPS to sediment stability in the field should be re-addressed and the importance of bacterial assemblages recognized.

## Materials and Methods

### Bacterial cultures

Subsurface sediment was sampled to a depth of 5–10 mm from an intertidal mudflat in the Eden estuary located in the southeast of Scotland (56°22′N, 2°51′W). One litre of 1 µm filtered seawater was mixed with the same volume of sediment and the sediment slurry was sonicated (Ultrasonic bath XB2 50–60 Hz) for 10 min. The sediment slurry was centrifuged twice (10 min, 6030 g, Mistral 3000E, Sanyo, rotor 43122-105) to separate sediment (pellet) and bacteria (supernatant). The supernatants were further centrifuged (10 min, 17700 g, Sorval RC5B/C) and this time the supernatant was discarded, while the remaining pellet with associated bacteria was re-suspended and filtered through a 1.6 µm filter (glass microfiber filter, Fisherbrand MF100). The filter size was chosen to exclude the smallest expected microalgae from the Eden estuary. Equipment was acid-washed and microalgal contamination was checked regularly by epifluorescense microscopy. Standard nutrient broth (Fluka, Peptone 15 g l^−1^, yeast extract 3 g l^−1^, sodium chloride 6 g l^−1^, D (+) glucose 1 g l^−1^) was autoclaved and added (1∶3) to the filtered supernatant. The bacterial stock cultures were established in 200 ml Erlenmeyer flasks under constant aeration in the dark at room temperature (15°C) and fresh nutrient broth was added once a week during 2 weeks cultivation.

### Diatom cultures

Sediment surface samples (0–5 mm) were taken from the same location on the Eden estuary and were initially processed as described for the bacterial cultures. However, the remaining pellet was resuspended in F/2 culture media without the filtration step. To exclude bacteria, antibiotics were added (150 mg l^−1^ streptomycin, 20 mg l^−1^ chloramphenicol, final concentrations) and the effective exclusion of bacteria was confirmed regularly by epifluorescense microscopy. The microalgal cultures were incubated under constant temperature (15°C) and at ambient light conditions in the laboratory for 2 weeks with fresh nutrients added regularly [23].

### Experimental set-up

A 3 cm layer (minimum operation depth of the Cohesive Strength Meter, CSM) of 0.04–0.07 mm glass beads was placed in Rotilab deep-freeze boxes (208L ×208W ×94H in mm). Two litres of autoclaved seawater were carefully added to each box [18]. Bacteria and diatom cultures served as inocula to initiate biofilms on the non-cohesive artificial substratum (Ballotini balls, glass beads). The following treatments were established (six replicates of each): controls (C), bacterial cultures (B), diatom cultures (D), as well as mixed assemblages of bacteria and diatom cultures (BD). The controls containing only glass beads and seawater were regularly treated (once a week) with a mixture of antibiotics (150 mg l^−1^ streptomycin and 20 mg l^−1^ chloramphenicol, final concentrations) to prevent bacterial colonisation. The other boxes were initially inoculated from the stock cultures with 15 ml each for bacterial and diatom cultures, and 30 ml (15/15 ml, B/D) for the mixed cultures. All treatments were gently aerated and kept at constant temperature (15°C) over a period of 4 weeks. The diatoms and the mixed assemblages were illuminated at 220–250 µmol photons m^−2^ s^−1^ under a light/dark cycle of 10/14 h.

### Sampling

Sampling took place on every third day during the experiment. For each treatment, 3 boxes out of the 6 replicates were randomly selected and sampled in turns at each measurement. From each sample box, 4 sediment cores of 5 mm depth were taken with a cut-off syringe (10 mm diameter) to determine bacterial cell numbers, bacterial assemblage (2 cores for 2 fixation protocols) and EPS. For the treatments diatoms (D) and the mixed assemblage (BD), 2 additional cores were taken to determine chlorophyll *a* and the microphytobenthic species composition. For bacterial dividing rate, 1 additional sediment core (depth 10 mm) was taken from the box and the 3 cores per treatment pooled before analysis; all other sediment cores were processed individually.

### Bacterial enumeration by flow cytometry

Cores were fixed with 0.2 µm pre-filtered glutaraldehyde solution (1% final concentration) and bacteria were stained with Syto13 (Molecular Probes, 1∶2000 v: v, 1.2 µmol l^−1^ final concentration) for 15 min in the dark. The bacterial abundance was measured by flow cytometery (Becton Dickinson FACScan™ with a laser emitting at 488 nm). Fluorescent calibrated beads were added to some samples (PeakFlow™, 6 µm, 515 nm, Molecular Probes) to distinguish bacterial cells from debris and mineral particles. The acquisition of events was thus limited to a gate encompassing the bacterial cells by plotting the side light scatter (SSC) versus green fluorescence (FL1). Data were recorded until 10,000 events were acquired or after 60 sec of counting. The bacterial abundance was calculated by multiplying the acquisition rates (between 160 and 640 bacteria counted per sec) by the flow rate (fixed to 60 µl min^−1^).

### Bacterial division rate

Cores were incubated for 20 min immediately after sampling with [methyl-3H] thymidine (final concentration 300 nmol L-1, S.A., 50 Ci mmol-1) according to Fuhrman and Azam [44]. The incorporation of radioactive thymidine was stopped by adding 5 ml of 80% ethanol. All the samples were collected on a filter (0.2 µm) after the incubation time and washed several times with 80% ethanol and 5% trichloroacetic acid (TCA) to remove excess radioactivity. The filters (containing the bacteria and the sediment particles) were mixed with 5 ml of 0.5 mol L^−1^ HCl and incubated at 95°C over 16 h [45] allowing the settlement of the sediment particles and the solubilisation of the stained bacteria into the supernatant. A subsample of the supernatant was taken, cooled and mixed with 3 ml of the scintillation cocktail Ultima Gold MV. The bacterial division rate (cells cm^−3^ h^−1^) was calculated according to an internal standard quenching curve (Liquid scintillation analyzer “TRI-CARB 2000”) while assuming that 1 mol^−1^ incorporated thymidine is equivalent to the production of 2×10^18^ bacterial cells [46], [47]. The saturating concentration of ^3^H-thymidine was chosen according to previous experiments in similar sediments. The thymidine incorporation was shown to be linear under the range of chosen concentrations [48], [49]. For each replicate, the radioactivity of the samples was corrected against a blank which corresponded to the pre-fixed sediment cores submitted to the protocol described above.

### Bacterial assemblage/Fluorescence in situ Hybridization (FISH)

To determine bacterial community composition, two sediment cores were fixed overnight with 3.7% formaldehyde and 70% ethanol to account for the different permeability of gram negative and gram positive bacteria, respectively [50], [51].After incubation (using a horizontal mixer, Denley Spiramix 5; Denley-Tech Ltd, Sussex, UK) and centrifugation (5 min at 16060 g^−1^, Biofuge pico Centrifuge, Heraeus, Rotor 7500 3325), the samples were washed twice with PBS, then resuspended in 500 µl of PBS. Applying a comprehensive set of oligonucleotide probes, intact bacterial cells were hybridized aiming at selective parts of the 16S rRNA that are specific for bacterial groups at the domain, phylum, and subphylum level (Table 1). The procedure is described in more detail in [18], [19], [52]. The hybridization with a molar mixture of the probes EUB338, EUB338II, and EUB338III gave the total eubacterial counts, and the probe-specific counts were calculated against these values as percentages.

### Pigment analysis

Cores were transferred to a 15 ml Apex centrifuge tube to which 10 ml of 96% ethanol was added. The tubes containing the mixture were rotated for 24 h in the dark and at room temperature (20°C) by a horizontal rotator at a fixed speed of 50 rpm (Denley Spiramix 5). The samples were centrifuged for 10 min at 6030 g (Mistral 3000E). The chlorophyll *a* and pheophytin concentrations in the supernatant were measured according to the Bmepc guidelines [53], reading absorbance at 630, 647, 664 and 750 nm wavelength before and after acidification (Termo Biomate 5 spectrophotometer), respectively, according to [54]. Chlorophyll a and pheophytin concentrations were given as a proxy for microphytobenthic biomass and degradation products, respectively, as microgram per cubic centimeter (µg cm^−3^).

### Microphytobenthos assemblage

The cores were fixed in 4% glutaraldehyde and the species composition of the microalgal community was assessed within 10 subsamples per sample by light microscopy. The organic was removed in subsamples which were then embedded in Naphrax (refractive index nD  = 1.710) for precise determination of taxa. The following literature was used: [55], [56], [57], [58], [59], [60].

### EPS extraction and determination

EPS from the sediment cores were extracted in safety-lock Eppendorf caps by adding 2 ml of distilled water (extractant). The samples were continuously rotated for 1.5 h, by a horizontal mixer (Denley Spiramix 5) at room temperature (20°C). After centrifugation (6030 g, 10 min, Mistral 3000E Sanyo, rotor 43122-105) the supernatant containing the water-extractable (colloidal) EPS fraction was pipetted into a new Eppendorf and mixed. Subsamples of this supernatant were analyzed in triplicates for carbohydrate and proteins following the Phenol Assay protocol [61], and the modified Lowry procedure [62]. For carbohydrates analysis, 200 µl phenol (5%) then 1 ml sulphuric acid (98%) were added to 200 µl supernatant. The samples were incubated for 35 min at 30°C and the carbohydrate concentration was measured by spectrophotometer (CECIL CE3021) at a wavelength of 488 nm [61], [63]. For protein analysis, 250 µl supernatant was incubated for 15 min with 250 µl of 2% sodium dodecyl sulphate salt (SDS) and 700 µl of chemical reagent 4 (Reagent 1∶143 mM NaOH, 270 mM Na_2_CO_3_, Reagent 2∶57 mM CuSO_4_, Reagent 3∶124 mM Na-tatrate, Reagent 4: a mixture of Reagent 1, 2 and 3 in a ratio of 100∶1∶1) and incubated for a further 45 min at 30°C with Folin reagent (diluted with distilled water 5∶6) [62], [63]. The protein concentration was measured by spectrophotometer (CECIL CE3021) at a wavelength of 750 nm. The carbohydrates and proteins concentrations are given in microgram per cubic centimeter (µg cm^−3^).

### Cohesive Strength Meter (CSM) measurements

The substratum stability was determined using the CSM [64]. A sequence of perpendicular water jets are fired at the test surface in the water-filled test chamber (30 mm in diameter), from a known height. The velocity of the jet pulses are increased until the bed fails [65] and sediment is resuspended. The CSM system records changes in transmission above the bed and a 10% drop in transmission from the original undisturbed bed is taken as the indication of resuspension and bed failure [65], [66]; The CSM program “Fine 1” offers a gradual increase in pressure steps over time and thus was most appropriate for the expected low range of stability. The relative substratum stability was expressed as stagnation pressure at the bed surface (Nm^−2^) causing a 10% decrease in transmission and measured at regularly intervals over the experimental period (7 times in 4 weeks).

### Magnetic Particle Induction (MagPI) measurements

Mechanical properties of the biofilms were studied with a new method based on the magnetic attraction of specially-produced test particles (Magnetic Particle Induction; MagPI [67]). This method is suitable for sensitive recording of changes of the surface adhesion of sediments or biofilms. Briefly, a known volume of ferromagnetic fluorescent particles (Partrac Ltd, UK, 180–250 µm) were spread onto a defined area of the sediment surface. The particles were then recaptured by an overlying electromagnet and the force (magnetic flux) needed to retrieve the particles was determined as a measure of the retentive capacity of the substratum, a proxy for adhesion. The electromagnetic force applied was finely controlled by a precision power supply (Rapid 5000 variable power supply) and the particle movements were precisely monitored at each increment of voltage/current. The MagPI was calibrated using a Hall probe and the results are given in mTesla [67]. The mechanical properties of the biofilm were studied in parallel to the CSM measurements over the experimental period of 4 weeks.

### Statistics

The data violated assumptions of normality and homogeneity of variance (visual assessment of the frequency histogram and normal plot, Kolmogorov-Smirnov and Barlett tests), thus differences between treatments were assessed using a non-parametric Kruskal-Wallis (χ^2^) test (KW), followed by the non-parametric Student-Newman-Keuls (SNK) test to correct for multiple comparisons.
